# Immunohistochemical Study of the Tumor Immune Microenvironment in Laryngeal Squamous Cell Carcinoma and Its Prognostic Implications

**DOI:** 10.3390/diagnostics16101431

**Published:** 2026-05-08

**Authors:** Mihaela-Iuliana Sirbu, Flavia Zara, Raluca Maria Closca, Marina Rakitovan, Antonia Armega-Anghelescu, Alexandru Cristian Cindrea, Ovidiu-Alexandru Mederle, Marcela-Maria Labadi, Nicolae-Constantin Balica

**Affiliations:** 1 Department of Ear-Nose-Throat, University of Medicine and Pharmacy “Victor Babes”, 300041 Timisoara, Romania; mihaela.sirbu@umft.ro (M.-I.S.);; 2 Department of Microscopic Morphology, University of Medicine and Pharmacy “Victor Babes”, 300041 Timisoara, Romania; 3 Department of Pathology, Emergency City Hospital, 300254 Timisoara, Romania; 4 Oro-Maxillo-Facial Surgery Clinic, Emergency City Hospital, 300062 Timisoara, Romania; 5 Doctoral School, Faculty of General Medicine, University of Medicine and Pharmacy “Victor Babes”, 300041 Timisoara, Romania; 6 Emergency Department, Emergency Clinical Municipal Hospital, 300254 Timisoara, Romania; mederle.ovidiu@umft.ro; 7 Emergency Discipline, Department of Surgery, University of Medicine and Pharmacy “Victor Babes”, 300041 Timisoara, Romania; 8 “Louis Țurcanu” Children’s Emergency Clinical Hospital, 300011 Timisoara, Romania

**Keywords:** laryngeal squamous cell carcinoma, tumor immune microenvironment, immunohistochemistry

## Abstract

**Background/Objectives**: Laryngeal squamous cell carcinoma exhibits marked heterogeneity in clinical behavior, which cannot be fully explained by conventional histopathological parameters alone. Increasing evidence highlights the pivotal role of the tumor immune microenvironment in modulating tumor progression and patient prognosis. **Methods**: The study group had 82 patients with laryngeal squamous cell carcinoma. A panel of immunohistochemical markers was chosen to identify and quantify key immune cell populations and immune-related components within the tumor immune microenvironment. Semiquantitative evaluation of immune infiltrates was conducted, with particular emphasis on their density and relative distribution across intratumoral and stromal compartments. **Results**: Based on the resulting immunophenotypic profiles, cases were categorized into three distinct immune patterns: an active immune type, defined by a prominent and dense inflammatory infiltrate; a mixed type, exhibiting intermediate and heterogeneous immune characteristics; and an immunosuppressive type, characterized by reduced effector immune cell infiltration and a predominance of immunoregulatory elements. Statistical analysis demonstrated significant correlations between these immune patterns and patient survival outcomes. **Conclusions**: The present study aimed to characterize the immune landscape of laryngeal squamous cell carcinoma using immunohistochemical markers and to evaluate its prognostic significance.

## 1. Introduction

Laryngeal cancer constitutes a significant proportion of malignant head and neck tumors, with a high impact on morbidity and mortality. Its relative frequency varies depending on geographical region. In developed countries, laryngeal cancer accounts for approximately 20–30% of malignant head and neck tumors, with higher proportions, reaching 30–40%, being observed in parts of Eastern and Southern Europe. Furthermore, Central and Eastern Europe has some of the highest incidence rates worldwide, estimated at 8–12 cases per 100,000 annually [1,2]. They may occur at the supraglottic, glottic, or subglottic levels, with each location having distinct staging particularities [3].

According to the American Cancer Society, in 2026, there will be an estimated 12,290 new cases of laryngeal cancer in the United States, including 9730 in men and 2560 in women. At the same time, the estimated number of deaths is 3960, including 3180 in men and 780 in women [4]. Compared to 2025, when 13,020 new cases and 3910 deaths were reported, these data indicate a slight decline in incidence, while mortality remains relatively unchanged, predominantly affecting males [5].

From a histopathological perspective, squamous cell carcinoma (SCC), arising from the epithelium of the laryngeal mucosa, accounts for the majority of malignant laryngeal neoplasms, exceeding 95% of all cases worldwide [6]. Its development is the result of a complex oncogenic process, mainly determined by prolonged exposure to carcinogens. In this context, the main etiological factors involved are long-term smoking and excessive alcohol consumption, along with other related factors, such as opium addiction, viral infections (HPV and EBV), exposure to environmental pollutants, including Agent Orange, changes in the microbiome, genetic susceptibility, and other environmental factors [7,8,9].

Head and neck cancers, including laryngeal squamous cell carcinoma (LSCC), are staged according to the TNM system established by the American Joint Committee on Cancer (AJCC), which serves as the standard framework for prognostic evaluation and treatment planning. In addition, the NCCN guidelines offer evidence-based recommendations for diagnosis, surgical and nonsurgical management, and follow-up, highlighting the critical role of precise staging and risk assessment in optimizing patient outcomes.

The main purpose of squamous cell carcinoma of the larynx treatment is to preserve the essential functions of the larynx as much as possible, thus maintaining a high survival rate and quality of life for the patient. In early-stage LSCC, definitive radiotherapy or conservative surgery are the recommended treatment options, with the choices guided by tumor location and anticipated functional outcomes. In contrast, in locally advanced disease, concurrent chemo- and radiotherapy are recommended for organ preservation when possible, while total laryngectomy, with or without postoperative therapy, is indicated for extensive disease or poor response. For recurrent or metastatic LSCC, systemic treatments, including platinum-based chemotherapy, immunotherapy, and targeted therapy agents, are used [10,11].

In this context, therapeutic approaches based on the tumor immune microenvironment (TME) have gained considerable interest in recent years, demonstrating significant clinical improvements in LSCC, while also being essential for a comprehensive understanding of the mechanisms involved in tumor progression, as well as for identifying new therapeutic and diagnostic strategies for the treatment of this cancer [12]. The TME represents a complex cellular environment that coexists with tumor cells and supports them in the process of progression towards malignancy, consisting of innate and adaptive immune cells, macrophages, stromal components, and elements of the extracellular matrix. This microenvironment underlies metabolic remodeling, including alterations in lipid, glucose, and amino acid metabolism, which promote tumor growth, survival, and resistance to treatment [13,14]. In LSCC, the TME includes tumor cells and cancer stem cells. It also includes cancer-associated fibroblasts, tumor-infiltrating lymphocytes, tumor-associated macrophages, and dendritic cells. In addition to cellular components, the TME also includes non-cellular elements such as cytokines, chemokines, growth factors, and non-coding RNAs, which all contribute to angiogenesis, immunosuppression, and tumor progression. These interactions are associated with important genetic and molecular alterations, with a direct impact on prognosis and therapeutic options in LSCC [14].

While previous studies described the prognostic significance of individual tumor-infiltrating immune cells in LSCC, the present study focuses on integrating these components into three distinct immune patterns. The novelty of this work lies not in the analysis of single markers per se but in the identification of immune pattern-based subtypes, which provide a more comprehensive and descriptive characterization of the tumor immune microenvironment and its prognostic heterogeneity.

The aim of this study was to characterize and quantify the cellular components of the TME in LSCC, using immunohistochemical analysis, and to correlate these findings with clinicopathological parameters and patient prognosis.

## 2. Materials and Methods

### 2.1. Patient Selection and Inclusion Criteria

This retrospective study included 82 patients diagnosed with laryngeal squamous cell carcinoma who were admitted to the Otorhinolaryngology Department of the Timisoara City Emergency Hospital between 1 January and 31 December 2018. These cases were selected for immunohistochemical analysis. All patients were subsequently followed for an 8-year period (January 2018 to December 2025) for oncological outcomes, including disease progression, recurrence, and the development of metastases. Survival data were obtained from the Central Population Registry database.

Inclusion criteria were as follows: patients over 18 years of age with initial presentation of a primary laryngeal tumor, histopathologically confirmed squamous cell carcinoma, complete clinical and imaging data, adequate and available paraffin blocks for immunohistochemical analysis, and a documented history of tobacco smoking and alcohol consumption.

**Figure 1:**
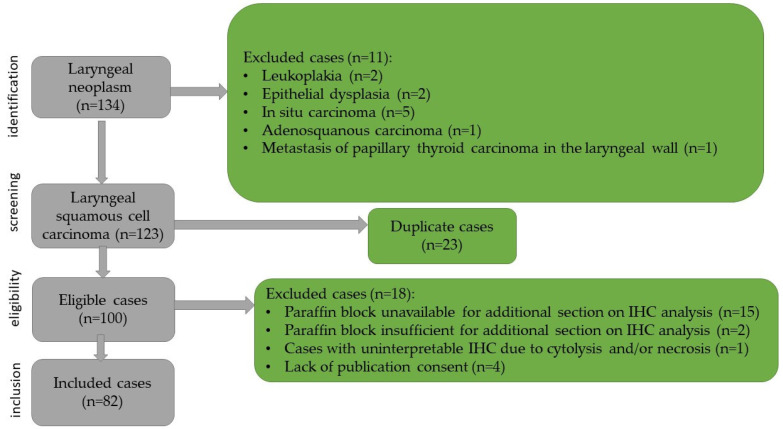
Flowchart of the case selection.

Exclusion criteria included cases with only a presumptive clinical diagnosis of laryngeal neoplasm without histopathological confirmation (e.g., leukoplakic lesions, epithelial dysplasia, or carcinoma in situ), other malignant laryngeal tumors such as adenosquamous carcinoma or metastatic lesions, duplicate cases, unavailable or insufficient paraffin blocks for additional sections or immunohistochemical analysis, patients under 18 years of age, and the lack of consent for publication. Additionally, prior radiotherapy to the head and neck region and recurrent tumors were considered exclusion criteria. The case selection process and exclusion criteria are presented in Figure 1.

### 2.2. Ethical Considerations

This study was conducted in accordance with Romanian legislation and the ethical principles of the Declaration of Helsinki. Informed consent was obtained from all of the patients, and the accompanying biopsy consent documentation was verified. Histological slide preparation followed the recommendations of the Ministry of Health and established international protocols. Ethical approval was granted by the Scientific Research Ethics Committee of the “Victor Babes” University of Medicine and Pharmacy, Timisoara (94/2 May 2022 rev 2024).

### 2.3. Laboratory Technique

The harvested specimens were fixed in 4% buffered formaldehyde. Sections measuring 4 μm in thickness were obtained using a semi-automated Leica RM2235 rotary microtome (Leica Biosystems, Nussloch, Germany) and mounted on SuperFrost™ glass slides (St. Louis, MO, USA). Routine hematoxylin–eosin staining was used for morphological evaluation. For each tumor, the following histopathological features were assessed: histological grade, keratinization, inflammatory response, perineural and/or angiolymphatic invasion, tumor budding, status of the resection margins, and the presence or absence of lymph node metastases.

Immunohistochemistry was performed for the assessment and quantification of the tumor immune microenvironment, using specific antibodies such as anti-p16, anti-CD20, anti-CD3, anti-CD4, anti-CD8, anti-CD68, anti-CD1a, and anti-CD117. All immunohistochemical procedures were carried out using the Leica Bond-Max automated staining system (Leica Biosystems Melbourne Pty Ltd., Waverley, Australia), in accordance with the manufacturer’s standardized protocols. All antibodies and reagents used for immunohistochemical staining were obtained from Leica Biosystems (Newcastle, UK). Detailed information on the immunohistochemical reagents, including antibody type, clone, and dilution, is provided in Table 1. Immunohistochemical staining was performed on sequential sections, each measuring 2 μm in thickness, with each tumor section stained using a single primary antibody.

The slides were evaluated independently by two experienced pathologists, who examined 10 high-power fields at 40× magnification for each section. Any interobserver discrepancies were resolved through joint review and consensus. Tumor grading was performed based on the nucleus–cytoplasm ratio, nuclear morphology, mitotic index, severity of atypia, and degree of keratinization, in three categories: G1—well differentiated; G2—moderately differentiated; and G3—poorly differentiated. Tumor budding was determined by reporting the counted clusters of one to five malignant cells at the invasion front, and tumors were stratified into low (≤4 buds) and high (>4 buds) groups. TNM staging of tumors was performed according to the AJCC 8th edition.

The tumor immune microenvironment was evaluated using a semiquantitative scoring system ranging from 0 to 3, defined as follows: score 0, ≤9 positive cells; score 1, 10–49 positive cells; score 2, 50–99 positive cells; and score 3, >100 positive cells per 10 high-power fields. Immunotypes were assigned based on a morphological and immunohistochemical evaluation of immune cell density using hematoxylin–eosin staining and immunohistochemical markers such as CD3, CD4, CD8, CD20, CD68, CD1a, and CD117. Both intratumoral and stromal compartments were considered. Cases were classified according to predefined criteria reflecting the relative abundance and distribution of immune populations into 3 patterns: immunotype A (active immune type): high density of effector T cells (CD3+, CD8+) and B cells (CD20+) predominantly intratumorally; immunotype B (mixed type): intermediate and heterogeneous immune cell densities across compartments; immunotype C (immunosuppressive type): low effector cell density with predominance of immunoregulatory components.

Formal thresholds for each marker were applied as described in Table 2. The assignment combined immunoscores and morphological assessment, ensuring a reproducible approach.

For the p16 protein, a positive reaction was considered to be nuclear and/or cytoplasmic immunostaining of high intensity in more than 70% of tumor cells.

### 2.4. Objectives

The first aim of this study was to identify and quantify the cellular components of the tumor immune microenvironment, and the second aim was to assess potential statistical correlations between the immune cells within the tumor microenvironment and various histopathological parameters with prognostic or predictive value.

### 2.5. Statistical Plan

Data analysis and graphical representation were performed using R v4.5.1, R Foundation for Statistical Computing, Vienna, Austria, using the following packages: *dplyr*, *readxl*, *stringr*, *janitor*, *lubridate*, *forcats*, *tidyr*, and *survival*.

The age of the patients was first assessed for normality using the Shapiro–Wilk test. Age was summarized as median and interquartile range (IQR) and compared between survivors and deceased patients using the Wilcoxon rank-sum test. Categorical variables were summarized as absolute counts and percentages and compared between groups using the Pearson chi-square test or Fisher’s exact test when expected cell counts were small.

Overall survival (OS) was defined from the date of diagnosis to the date of death or censoring at the study end date. Patients with inconsistent survival coding (non-survivors without a recorded date of death) were excluded from survival analyses. OS was estimated using the Kaplan–Meier method and compared across immunotype groups (A, B, and C) using the log-rank test. The association between immunotype and OS was further evaluated using Cox proportional hazards regression, first in univariable analysis and then in adjusted models including selected clinicopathologic covariates (pT, pN, vascular/lymphatic invasion, and histological grade, where available). Results from Cox models were reported as hazard ratios (HRs) with 95% confidence intervals (CIs). The proportional hazards assumption was checked using Schoenfeld residuals. Events per parameter were calculated for the adjusted Cox model. Internal validation was conducted by bootstrap resampling, and model discrimination was summarized using the apparent and optimism-corrected concordance index. In addition, a ridge-penalized Cox regression was fitted as a sensitivity analysis to evaluate whether the association between immunotype and overall survival remained directionally consistent after coefficient shrinkage.

All *p* values reported are two-tailed, and *p* values < 0.05 were considered statistically significant.

## 3. Results

### 3.1. Clinical and Demographic Data

This study included 82 cases of laryngeal squamous cell carcinoma, with a median age at diagnosis of 70 years and a predominance of male patients. Among men, the highest incidence occurred in the 6th and 7th decades (*n* = 63, 77%), while in women it was observed in the 6th decade (*n* = 2, 2%). Patient histories revealed that 68.3% (*n* = 56) had smoked at least one pack of cigarettes per day over the past 10 years, and 16% (*n* = 13) reported concurrent chronic alcohol consumption. In the present cohort of laryngeal squamous cell carcinoma, the majority of tumors were located on the vocal cords (*n* = 71, 86.6%), reflecting the most common anatomical site of disease. Supraglottic tumors were observed in eight cases (9.8%), while subglottic tumors were rare, comprising only three cases (3.7%)

The patients were diagnosed by microscopic examination of laryngeal biopsies. After the histopathological diagnosis, all patients were monitored through oncological follow-up and received chemotherapy and/or radiotherapy. In total, 39% (*n* = 32) were in early and limited stages of the disease (T1 and T2) and had biopsy or CO_2_ laser cordectomy, while 61% (*n* = 50) were in advanced stages (T3 and T4) and had total laryngectomy. For 18% of the patients (*n* = 15), locoregional lymph node dissection was also performed. Over half of them (*n* = 49, 60%) died within the next five years.

Additional clinical and epidemiological information is presented in Table 3.

### 3.2. Morphological Features

Microscopic examination using hematoxylin–eosin staining showed that most laryngeal carcinomas (*n* = 82, 93%) were of the conventional type of squamous cell carcinoma, while four cases were of the basaloid type (*n* = 4, 5%), and one case each was of the verrucous and papillary subtypes of squamous cell carcinoma (*n* = 1, 1%). The tumors were predominantly moderately differentiated G2 (88%, *n* = 72) or poorly differentiated G3 (9%, *n* = 7), with only 3% (*n* = 3) classified as well-differentiated G1. Most tumors were of the non-keratinized subtype (68%, *n* = 56), while the remaining 32% (*n* = 26) were keratinized, exhibiting central keratin pearl formation. Budding tumor with the presence of clusters of one to five tumor cells at the invasion front was identified in 29% of cases (*n* = 24), all of which were high-stage, T3 and T4, while almost half of the patients had angiolymphatic invasion (46%, *n* = 38) and only one-fifth had perineural invasion (21%, *n* = 17) (Table 4).

**Figure 2:**
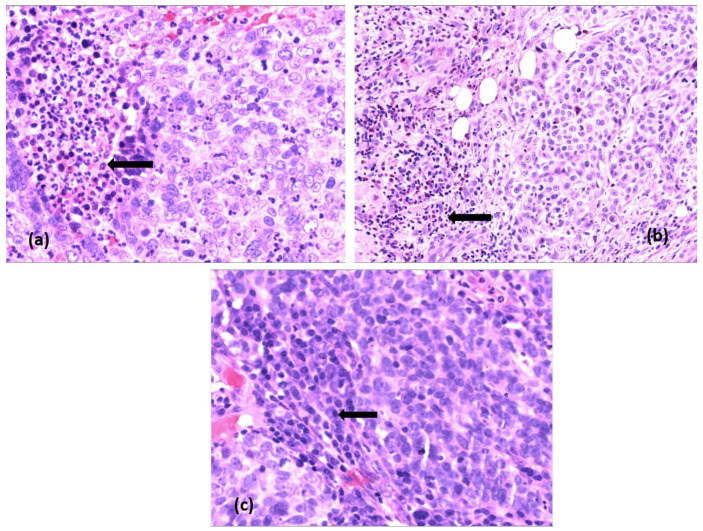
Tumor immune microenvironment of LSCC—morphological aspects in hematoxylin–eosin staining, ob.40×: (**a**) neutrophilic granulocytes, with suppurative areas (arrow); (**b**) eosinophils (arrow); (**c**) plasma cells (arrow).

The presence of neutrophil granulocytes was observed in the peritumoral areas, in only 13 cases (16%), with a variable score, between 0 and 3, both in immunotypes A, B and C. However, in tumors with immunotype A, neutrophil granulocytes had slightly higher scores of 2 and 3, with the formation of suppurative microfoci. In contrast, plasma cells and eosinophilic granulocytes had a high density in intratumoral areas, with scores of 2 and 3, predominantly in type A tumors (Figure 2).

### 3.3. Immunohistochemical Features

#### 3.3.1. Immunohistochemical Features of p16-Negative Tumors

The majority of laryngeal carcinomas (*n* = 77, 94%) were p16-negative, with moderate to weak intensity reaction in less than 80% of tumor cells.

**Figure 3:**
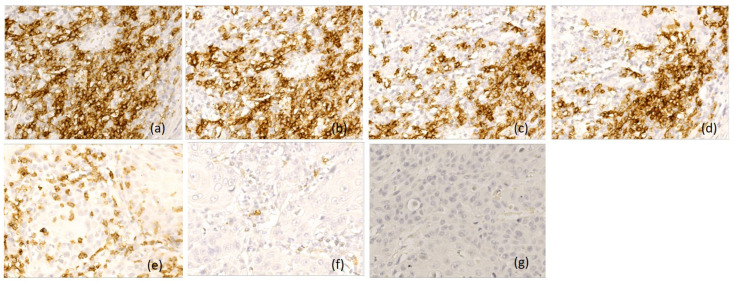
Representative immunohistochemical profile of LSCC with immunotype A, ob.40×: (**a**) CD 20+ B lymphocytes, immunoscore 3; (**b**) CD3+ T lymphocytes, immunoscore 3; (**c**) CD4+ T lymphocytes, immunoscore 2; (**d**) CD8+ T lymphocytes, immunoscore 3; (**e**) CD1a+ dendritic cells, immunoscore 3; (**f**) CD68+ macrophages, immunoscore 0; (**g**) CD117+ mast cells, immunoscore 0. Images are representative of the dominant immune pattern.

Based on the immunoscores, we classified p16-negative tumors into three patterns. Type A tumor (*n* = 21, 27%) had an active immune tumor microenvironment, with increased density of CD20-positive B lymphocytes, CD3-, CD4- and CD8-positive T lymphocytes, and CD1a-positive antigen-presenting dendritic cells (score 3 and 2), associated with rare or absent CD68-positive macrophages and CD117-positive mast cells (score 0). B lymphocytes were distributed both peritumorally and intratumorally, while T lymphocytes were predominantly in tumor stroma, along with neutrophil and eosinophil granulocytes. Antigen-presenting dendritic cells had an increased density in the peritumoral areas and in the adjacent mucosa (Figure 3).

**Figure 4:**
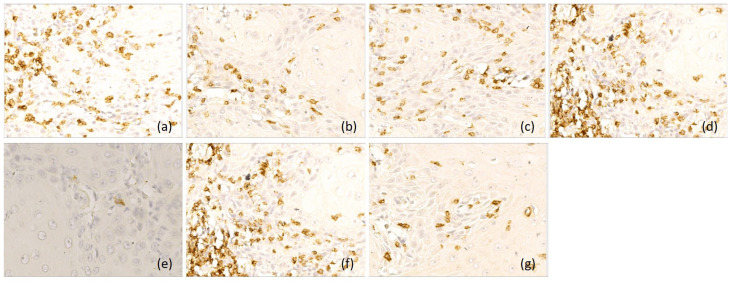
Representative immunohistochemical profile of LSCC with immunotype B, ob.40×: (**a**) CD 20+ B lymphocytes, immunoscore 2; (**b**) CD3+ T lymphocytes, immunoscore 1; (**c**) CD4+ T lymphocytes, immunoscore 1; (**d**) CD8+ T lymphocytes, immunoscore 2; (**e**) CD1a+ dendritic cells, immunoscore 1; (**f**) CD68+ macrophages, immunoscore 2; (**g**) CD117+ mast cells, immunoscore 1. Images are representative of the dominant immune pattern.

Type B laryngeal carcinomas (*n* = 17, 22%) were characterized by a mixed immune microenvironment, with variable density of peritumoral and intratumoral CD8-positive T lymphocytes (score 0–3) and moderate density of CD68-positive macrophages (score 1 and 2) in peritumoral areas (Figure 4).

**Figure 5:**
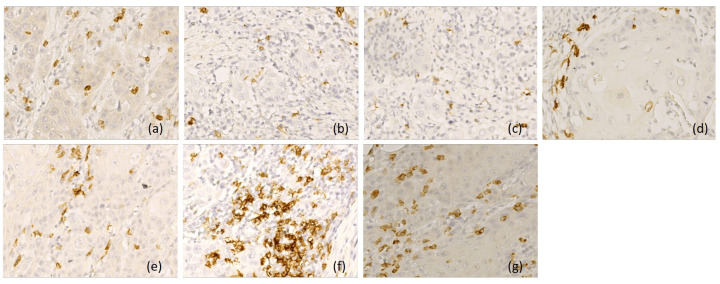
Representative immunohistochemical profile of LSCC with immunotype C, ob.40×: (**a**) CD 20+ B lymphocytes, immunoscore 1; (**b**) CD3+ T lymphocytes, immunoscore 0; (**c**) CD4+ T lymphocytes, immunoscore 0; (**d**) CD8+ T lymphocytes, immunoscore 1; (**e**) CD1a+ dendritic cells, immunoscore 1; (**f**) CD68+ macrophages, immunoscore 3; (**g**) CD117+ mast cells, immunoscore 2. Images are representative of the dominant immune pattern.

Type C tumor (*n* = 39, 51%) showed immunosuppressive microambient, with CD3-, CD4- and CD8-positive T lymphocytes and CD1a-positive antigen-presenting dendritic cells absent or rare (score 0 and 1), but an increased density in CD68-positive macrophages (score 3) and CD117-positive mast cells (scores 2 and 3), especially in peritumoral areas (Figure 5).

No significant association was identified between age and immunotype distribution (Kruskal–Wallis test, *p =* 0.579). Median age was 71.0 (64.0–76.0) years in immunotype A, 69.0 (64.2–73.2) years in immunotype B, and 68.0 (65.0–74.0) years in immunotype C. Sex was also not significantly associated with immunotype distribution (Fisher’s exact test, *p =* 0.457). No significant association was identified between smoking status, alcohol consumption and immunotype distribution (*p* = 0.650; *p* = 0.460).

No significant association was identified between histopathological subtype group and immunotype distribution (Fisher’s exact test, *p* = 0.209). In contrast, tumor stage was significantly associated with immunotype distribution. For the simplified pT classification, immunotype A was more frequently observed in T1 tumors (52.0%), whereas immunotypes B and C were predominantly associated with advanced tumors classified as T3–4 (65.0% and 81.1%, respectively; Fisher’s exact test, *p* < 0.001). This pattern remained significant when tumors were grouped as early-stage (T1–2) versus advanced-stage (T3–4), with immunotype A predominating in early-stage tumors (72.0%), while immunotypes B and C were more frequent in advanced-stage disease (65.0% and 81.1%, respectively; chi-square test, *p* < 0.001). No significant association was identified between immunotype and nodal status, either for pN grouped as N0 versus N+ (Fisher’s exact test, *p* = 0.315) or for the detailed pN categories (Fisher’s exact test, *p* = 0.417).

An association was observed between immunotype and histological grade when grade was analyzed in the original three-category format (G1, G2, and G3; Fisher’s exact test, *p* = 0.049). Immunotype A consisted exclusively of G2 tumors, whereas G1 tumors were identified only in immunotype C, and G3 tumors were present in immunotypes B and C. However, this association was not confirmed in sensitivity analyses using grouped grade categories (G1–2 vs. G3; Fisher’s exact test, *p* = 0.054) or ordinal testing (Kruskal–Wallis test, *p* = 0.135).

#### 3.3.2. Immunohistochemical Features of p16-Positive Tumors

**Figure 6:**
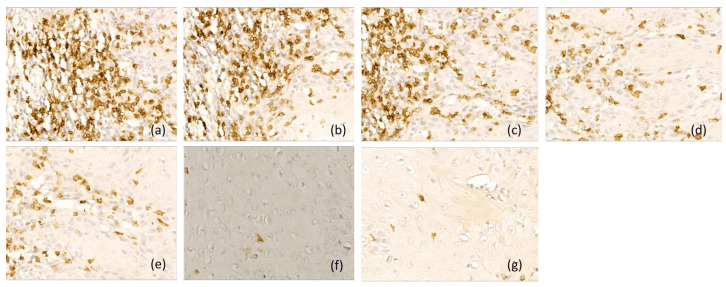
Representative immunohistochemical profile of p16-positive LSCC, ob.40×: (**a**) CD 20+ B lymphocytes, immunoscore 3; (**b**) CD3+ T lymphocytes, immunoscore 3; (**c**) CD4+ T lymphocytes, immunoscore 2; (**d**) CD8+ T lymphocytes, immunoscore 2; (**e**) CD1a+ dendritic cells, immunoscore 2; (**f**) CD68+ macrophages, immunoscore 0; (**g**) CD117+ mast cells, immunoscore 0. Images are representative of the dominant immune pattern.

Only 6% of tumors (*n* = 5) were p16-positive, with intense and diffuse reaction in 80–100% of tumor cells, showing both nuclear and cytoplasmic immunostaining. p16-positive carcinomas (*n* = 5, 6%) were type A and B, with high density of peri- and intratumoral CD20-positive B lymphocytes and CD3-, CD4- and CD8-positive T lymphocytes, located predominantly intratumorally. In this cohort, these immunohistochemical features overlapped with those observed in a p16-negative type A tumor (Figure 6).

The immunohistochemical profile of the tumor immune microenvironment for both p16-positive and p16-negative tumors is shown in Table 5.

Smoking status and alcoholism were not significantly associated with any of the semiquantitative scores of the evaluated immune markers (all *p*-values > 0.05).

A significant association was identified between p16 status and immunotype distribution (Fisher’s exact test, *p* = 0.003). All p16-positive tumors (*n* = 5) were classified as immunotype A, whereas no p16-positive cases were observed in immunotypes B or C. No significant association was found between p16 status and smoking status (Fisher’s exact test, *p* = 1.000), nodal status (Fisher’s exact test, *p* = 1.000), histological grade (Fisher’s exact test, *p* = 1.000), or sex (Fisher’s exact test, *p* = 1.000). A non-significant trend toward lower tumor stage was observed in p16-positive cases, both for the grouped comparison T1–2 versus T3–4 (Fisher’s exact test, *p* = 0.073) and for the simplified pT classification (Fisher’s exact test, *p* = 0.081).

### 3.4. Survival Analysis

**Figure 7:**
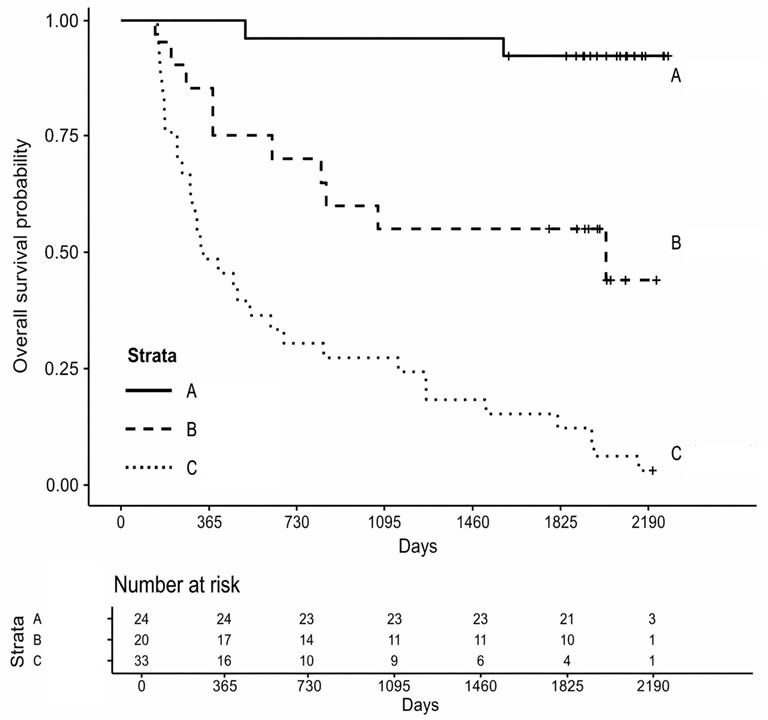
Kaplan–Meier overall survival curves stratified by immunotype (A, B, C). Survival differed significantly between immunotypes (log-rank *p* < 0.001).

Table 6 presents the OS at 1,2 and 5 years for each immunotype. A total of 77 patients were included in the survival analysis (Figure 7), with 44 deaths observed. Five patients were excluded from this analysis because no date of death was recorded. Kaplan–Meier curves demonstrated significantly different OS across immunotypes A, B, and C (log-rank *p* < 0.001), with immunotype A showing the most favorable survival and immunotype C the poorest survival. In multivariable Cox regression adjusted for pT and pN categories, VL, and grade, immunotype remained independently associated with OS. Compared with immunotype A, immunotype B was associated with a significantly increased hazard of death (HR 7.39; 95% CI 1.50–36.33; *p* = 0.0138), and immunotype C showed an even higher hazard (HR 24.79; 95% CI 5.55–110.81; *p* < 0.001). The additional covariates included in the model were not statistically significant (all *p* > 0.05). These associations remained stable in parsimonious sensitivity models adjusting separately for key clinicopathologic factors, with HRs for immunotypes B and C consistently in the same range.

Because the number of events relative to the number of model parameters was modest, additional internal validation analyses were performed to assess model stability. The multivariable Cox model included 44 deaths and 7 parameters, corresponding to an events-per-parameter ratio of 6.29. Bootstrap resampling showed that the direction of the association between immunotype and overall survival was preserved, although the bootstrap intervals were wide, indicating limited precision in the magnitude of the hazard ratio estimates. In a ridge-penalized Cox regression, immunotype remained associated with overall survival, with hazard ratios of 7.65 (95% CI 1.59–36.75; *p* = 0.011) for immunotype B and 26.31 (95% CI 6.00–115.43; *p* < 0.001) for immunotype C, compared with immunotype A. The apparent concordance index of the model was 0.782, with an optimism-corrected concordance index of 0.749.

Survival differed significantly by immunotype within both early-stage (T1–2) and advanced-stage (T3–4) disease (log-rank *p* < 0.001 for both strata). In Cox regression, there was evidence of effect modification by stage: an interaction term between immunotype and stage group (T1–2 vs. T3–4) significantly improved model fit (likelihood ratio test *p* = 0.012), indicating that the prognostic impact of immunotype varied by stage.

## 4. Discussion

The progression of LSCC involves both genetic changes and remodeling of the TME, which promotes invasion, metastasis, and immune evasion. Recent studies have shown that genetic and cellular factors, including lymphocytes, macrophages, stromal cells, and immune molecules, influence tumor aggressiveness and prognosis. In this context, characterizing the composition and distribution of the immune infiltrate has become essential for understanding progression and prognosis, and identifying therapeutic strategies [14].

LSCC is one of the most common types of head and neck cancers (HNCs), directly associated with tobacco and alcohol consumption. In recent years, the incidence of tobacco-related cancers has decreased, while HPV infection has increased significantly, particularly at young ages, due to types 16, 18, and 31 [15]. This p16 protein is widely used as a surrogate marker of HPV infection, playing a role in inhibiting cell cycle progression [16] and being associated in many studies with a favorable prognosis in laryngeal cancer [17,18]. Although p16 is recognized as a surrogate marker for HPV and has been associated with a favorable prognosis, its implication in LSCC and its association with the TME remain poorly understood.

In this study, we analyzed the TME of 82 patients with LSCC, using an immunohistochemical panel targeting key inflammatory and immune cell populations. The following immunohistochemical markers were applied to identify inflammatory cells: CD20 for B lymphocytes; CD3, CD4, and CD8 for T lymphocytes; CD68 for macrophages; CD1a for antigen-presenting dendritic cells; and CD117 for mast cells. Plasma cells, granulocytes, neutrophils, and eosinophils were identified based on histological staining. After quantifying the inflammatory cells, we correlated them with clinical and pathological parameters. Furthermore, we identified three distinct immune patterns, including active, intermediate, and immunosuppressive, all of them associated with patient prognosis. Together, these findings support the premise that the immune context represents a major determinant of LSCC progression and clinical outcome.

Several studies have demonstrated that increased density of tumor-infiltrating lymphocytes (TILs), particularly CD8+ T cells, has been associated with improved survival in patients with LSCC and other HNC. T cells recognize tumor-specific neoantigens and induce malignant cell lysis, and CD8+ infiltrates correlate with HLA class I expression on tumor cells, while HLA-I downregulation and low CD8+ density are associated with poor prognosis. Clinical studies, involving patients with HNSCC, have demonstrated that high levels of CD8+ TILs are associated with better disease-specific and overall survival, including in LSCC [19,20]. Also, further studies evaluating immune infiltrates in the TME of HNSCC have confirmed that increased TIL density is associated with improved patient survival. This is particularly observed in LSCC, where a more favorable prognosis has been associated with meta-analyses performed on different HNSCC subtypes, confirming the positive prognostic impact of CD3+ and CD8+ T cell infiltration [21,22]. Consistent with these observations, immune-active tumors (type A) within our cohort were characterized by increased densities of T lymphocytes, predominantly located within the tumoral compartment, and were associated with a more favorable prognosis.

Also, immunotype A tumors in our group showed a high density of CD20+ B lymphocytes. These play a role in antibody production and may contribute to antitumor immunity by presenting antigens and supporting CD8+ T cell responses within tertiary lymphoid structures. The association between CD20+ B lymphocytes and CD8+ T lymphocytes has been linked to improved survival in several tumors, indicating a possible complementary involvement in effective immune surveillance [23]. This observation is supported by previous studies that have shown that an increased density of CD20+ B lymphocytes and a higher density of tertiary lymphoid structures are most frequently found in patients responding to therapy, supporting the concept that B cell infiltration represents an active antitumor immune response [24]. Thus, the increased density of CD20+ B lymphocytes in immunotype A tumors may contribute to the significant improvement in survival rate observed in our group.

Dendritic cells are antigen-presenting cells with different subtypes depending on their origin, location, and phenotype. Derived from CD34+ precursors in the bone marrow, they migrate to peripheral tissues to capture antigens and then to lymphoid tissues to process and present them to T cells. In HNSCC, myeloid dendritic cells enhance immune responses, while plasmacytoid dendritic cells may promote immunosuppression and tolerance. Increased dendritic cell density in the HNSCC TME has been associated with a favorable prognosis, while inhibition of their function by tumors may contribute to reduced antitumor immunity and poor treatment efficacy [25,26]. Accordingly, current studies indicate that an increased number of myeloid dendritic cells correlates with a lower metastasis rate, a reduced recurrence, and an improved survival in patients with HNSCC [27,28]. Consistent with previous reports, increased CD1a-positive dendritic cell density was associated with a favorable prognosis in our study.

Macrophages originate from circulating monocytes and represent a heterogeneous cell population with high plasticity, capable of adopting either antitumor or pro-tumor phenotypes depending on local stimuli and tumor microenvironment conditions [29]. In many malignant tumors, the predominance of pro-tumor tumor-associated macrophages has been described in association with immunosuppressive microenvironments and tumor progression [30]. Increased secretion of growth factors and chemokines has also been reported in association with adverse prognostic features [25]. In the present study, immunotype C tumors were characterized by a high density of CD68+ macrophages. Previous studies in head and neck cancer have described an association between increased infiltration of CD68+ tumor-associated macrophages and aggressive tumor behavior, as well as poorer survival [25,31]. Consistent with these observations, in our cohort, immunotype C tumors exhibited both a higher density of CD68+ macrophages and reduced overall survival. However, these findings should be interpreted as correlative and do not allow conclusions regarding a causal role of macrophages in tumor progression, treatment resistance, or patient outcome [31].

Similarly, tumors classified as immunotype C exhibited an increased density of mast cells, which, in our cohort, was associated with higher mortality. Mast cells are present in the TME of many tumors, and their role in disease prognosis depends on the type of cancer. In esophageal, ovarian, and lymphomas, increased mast cell density has been associated with a favorable prognosis. In contrast, in other types of cancer, such as breast, gastric, and lung carcinoma, the presence of tumor-associated mast cells has been correlated with a more unfavorable outcome [32].

In head and neck squamous cell carcinoma, a systematic review by Tzorakoleftheraki et al. highlighted the variable prognostic impact of mast cells, with studies reporting both unfavorable and potentially favorable associations [33]. These differences have been discussed in relation to factors such as tumor location, microenvironmental context, and cellular interactions. In our cohort, the higher density of mast cells observed in immunotype C tumors occurred alongside reduced overall survival; however, this finding should be interpreted as a correlation and does not allow conclusions regarding a causal or mechanistic role of mast cells in tumor progression [34].

In addition to the major immune cell populations described above, other inflammatory components were also identified within the TME, including plasma cells, eosinophils, neutrophils, and granulocytes. Similarly, increased intratumoral plasma cells and eosinophils were associated with improved survival in our cohort, whereas neutrophils showed only a modest association with the outcome. Tumor-associated B cells, including plasma cells, have been reported in the literature in association with a favorable or neutral prognosis in several cancer types. Plasma cells may be components of tertiary lymphoid structures and have been described in relation to antitumor immune responses; however, the functional significance of these findings cannot be established based on the present data [35]. Eosinophils are mature leukocytes involved in regulating immune response and tumor progression. In several types of cancer, they have been associated with antitumor effects and improved survival, particularly in the context of immunotherapy, although in some hematological conditions, they indicate a poor prognosis. In treatment with immune checkpoint inhibitors, the absolute number of eosinophils has been correlated with both therapeutic response and adverse events [36]. Neutrophils or polymorphonuclear leukocytes are the main innate immune effector cells and, along with macrophages, represent the first line of defense at the site of infection and injury. They are involved in the adaptive immune response through antigen presentation and T cell activation, and through the release of lytic enzymes and reactive oxygen species, neutrophils can form extracellular traps [37].

Our findings highlight the practical value of integrating immune components into immune models, rather than focusing exclusively on individual markers. This approach provides a framework for descriptively assessing prognostic heterogeneity and complements existing studies on individual immune markers. We emphasize that the main contribution of this study is the integrative analysis of the tumor immune microenvironment, which can help stratify risk and generate hypotheses for future research, rather than establishing new individual biomarker associations.

This study is subject to several limitations that should be considered when interpreting the findings. The relatively small sample size, together with the retrospective design, may restrict the generalizability of the results and does not allow for the establishment of causal relationships. In particular, the limited number of p16-positive cases (*n* = 5) constrains the robustness of subgroup analyses and precludes reliable conclusions regarding this subset. The semiquantitative immunohistochemical evaluation of immune infiltrates may be influenced by interobserver variability and may not fully capture intratumoral heterogeneity. Furthermore, HPV status was assessed exclusively by immunohistochemistry, without validation through in situ hybridization, which could impact the accuracy of case classification. Therefore, these findings should be interpreted with caution, and further studies involving larger cohorts and complementary methodological approaches are required to substantiate these results.

## 5. Conclusions

Statistical analysis identified some correlations between immune patterns and patient survival outcomes within our cohort. The active immune type was more frequently observed in cases with a favorable prognosis, whereas the immunosuppressive type predominated in cases with reduced overall survival, and the mixed type showed intermediate features.

Our findings highlight patterns identified through immunohistochemical profiling of the tumor immune microenvironment in LSCC and may offer a descriptive framework for characterizing prognostic heterogeneity. Nevertheless, these observations remain exploratory and require validation in larger, independent cohorts before any clinical application can be considered.

## Figures and Tables

**Table 1:** Data related to the antibodies used for the immunohistochemical study.

Antibody	Substrate	Clone	Dilution	Cell Type
^1^ CD20	Monoclonal, mouse	L26	^2^ RTU	B Lymphocyte
^3^ CD3	Monoclonal, mouse	LN10	1:500	T lymphocyte
^4^ CD4	Monoclonal, mouse	4B12	1:100	T helper lymphocyte
^5^ CD8	Monoclonal, mouse	4B11	1:500	T cytotoxic lymphocyte
^6^ CD68	Monoclonal, mouse	514H12	1:100	Macrophage
^7^ CD1a	Monoclonal, mouse	MTB1	RTU	Dendritic cell
^8^ CD117	Monoclonal, rabbit	EP10	1:200	Mast cell
^9^ p16	Monoclonal, mouse	CS1	1:100	Tumor cells

^1^ CD20 (cluster of differentiation 20); ^2^ RTU (ready-to-use); ^3^ CD3 (cluster of differentiation 3); ^4^ CD4 (cluster of differentiation 4); ^5^ CD8 (cluster of differentiation 8); ^6^ CD68 (cluster of differentiation 68); ^7^ CD1a (cluster of differentiation 1a); ^8^ CD117 (cluster of differentiation 117); ^9^ p16 (inhibitor of cyclin-dependent kinase 4a).

**Table 2:** Immunotype classification criteria in LSCC.

Immunotype	Marker	Compartment	Score/Density	Assignment Criteria
A	CD20 (B cells)	Intra- and peritumoral	2–3	^1^ +++
	CD3 (T cells)	Peritumoral	2–3	+++
	CD4 (T helper cells)	Peritumoral	2–3	+++
	CD8 (cytotoxic T cells)	Peritumoral	2–3	+++
	CD1a (dendritic cells)	Peritumoral/Adjacent mucosa	2–3	+++
	CD68 (macrophages)	Intra- and peritumoral	0	^2^ −
	CD117 (mast cells)	Intra- and peritumoral	0	−
	Plasma cells	Intratumoral	2–3	+++
	Eosinophils	Intratumoral	2–3	+++
	Neutrophils	Peritumoral	2–3	+++, microsupurative areas
B	CD8 (T cells)	Intra- and peritumoral	0–3	Variable density across compartments
	CD68 (macrophages)	Peritumoral	1–2	^3^ ++
	B and T cells markers	Intra-/peritumoral	0–3	Heterogeneous/intermediate
C	CD3, CD4, and CD8 (T cells)	Intra-/peritumoral	0–1	−
	CD1a (dendritic cells)	Intra-/peritumoral	0–1	−
	CD68 (macrophages)	Peritumoral	3	+++
	CD117 (mast cells)	Peritumoral	2–3	+++
	Neutrophils	Peritumoral	0–3	Heterogeneous/intermediate
	Plasma cells, eosinophils	Intratumoral	0–1	^4^ +/−

^1^ +++ (high density); ^2^ − (rare or absent); ^3^ ++ (moderate density); ^4^ + (low density).

**Table 3:** Clinical and epidemiological characteristics of the analyzed tumors.

Variable	All Patients	Survivors	Deceased	*p*-Value
Age ^a^	70 (64.2–74)	70 (64–74)	69 (66–76)	0.596
Sex (male) ^b^	78 (95.1%)	32 (97%)	46 (93.9%)	0.645
Smoker ^b^	56 (68.3%)	24 (72.7%)	32 (65.3%)	0.479
Tumor site				
Vocal cords ^b^	71 (86.6%)	28 (84.9%)	43 (87.8%)	0.872
Supraglottic ^b^	8 (9.7%)	4 (12.1%)	4 (8.2%)	0.665
Subglottic ^b^	3 (3.7%)	1 (3%)	2 (4%)	0.578

^a^ median (interquartile range), Wilcoxon test. ^b^ absolute (relative value), either chi-square statistical test or Fisher’s exact.

**Table 4:** Microscopic aspects of the tumors analyzed.

Variable	All Patients	Survivors	Deceased	*p*-Value
Histological type				
Keratinized	27 (32.9%)	12 (36.4%)	15 (30.6%)	0.637
Non-keratinized	55 (67.1%)	21 (63.6%)	34 (69.4%)	
Histological grade				
Well differentiated	3 (3.7%)	–	3 (6.1%)	0.456
Moderately differentiated	71 (86.6%)	30 (90.9%)	41 (83.7%)	
Poorly differentiated	8 (9.7%)	3 (9.1%)	5 (10.2%)	
Tumor stage				
T1	21 (25.6%)	14 (42.4%)	7 (14.3%)	0.009 *
T2	11 (13.4%)	5 (15.2%)	6 (12.2%)	
T3 and T4	50 (61%)	14 (42.4%)	36 (73.5%)	
N0	72 (87.8%)	30 (90.9%)	42 (85.7%)	0.733
N1	10 (12.2%)	3 (9.1%)	7 (14.3%)	
Angiolymphatic invasion	38 (46.3%)	8 (24.2%)	30 (61.2%)	<0.001 **
Tumor budding	24 (29.3%)	1 (3%)	23 (46.9%)	<0.001 **
Perineural invasion	21 (25.6%)	4 (12.1%)	17 (34.7%)	0.022

* and **: statistically significant at *p* < 0.05 and *p* < 0.01.

**Table 5:** Immunohistochemical profile of the tumor immune microenvironment.

Variable	All Patients	Survivors	Deceased	*p*-Value
Tumor immunotype				
A	25 (30.5%)	22 (66.7%)	3 (6.1%)	<0.001
B	20 (24.4%)	10 (30.3%)	10 (20.4%)	
C	37 (45.1%)	1 (3%)	36 (73.5%)	
CD20+ B cell score	1 (0–2)	3 (2–3)	0 (0–1)	<0.001
CD3+ T cell score	2 (0–3)	3 (3–3)	0 (0–1)	<0.001
CD4+ T cell score	1 (0–2)	2 (2–3)	0 (0–1)	<0.001
CD8+ T cell score	1 (0–3)	3 (2–3)	0 (0–1)	<0.001
CD117+ mast cell score	2 (1–3)	0 (0–1)	3 (2–3)	<0.001
CD68+ macrophage score	2 (1–3)	1 (0–1)	3 (2–3)	<0.001
CD1a+ dendritic cell score	1 (0–2)	2 (2–3)	0 (0–1)	<0.001
Plasma cell score	0 (0–2)	2 (1–3)	0 (0–0)	<0.001
Neutrophil score	0 (0–0)	0 (0–1)	0 (0–0)	0.019
Eosinophil score	0 (0–2)	2 (1–2)	0 (0–0)	<0.001

**Table 6:** OS at 1 year, 2 years and 5 years following diagnosis.

Immunotype	1-Year OS	2-Year OS	5-Year OS
A	100% (100–100)	95.8% (88.2–100)	91.7% (81.3–100)
B	85% (70.7–100)	70% (52.5–93.3)	55% (37–81.8)
C	48.5% (34.1–68.9)	30.3% (18.1–50.8)	12.1% (4.8–30.4)

## Data Availability

The data that support the fundings on this study are available from the corresponding author upon reasonable request. Medical and personal data are not publicly available due to General Data Protection Regulation.

## Abbreviations

The following abbreviations are used in this manuscript:

SCC	Squamous Cell Carcinoma
HPV	Human Papillomavirus
EBV	Epstein–Barr Virus
LSCC	Laryngeal Squamous Cell Carcinoma
TNM	Tumor, Nodes, and Metastasis
AJCC	American Joint Committee on Cancer
NCCN	National Comprehensive Cancer Network
TME	TME Tumor Immune Microenvironment
CD20	Cluster of Differentiation 20
RTU	Ready-to-use
CD3	Cluster of Differentiation 3
CD4	Cluster of Differentiation 4
CD8	Cluster of Differentiation 8
CD68	Cluster of Differentiation 68
CD1a	Cluster of Differentiation 1a
CD117	Cluster of Differentiation 117
p16	Inhibitor of Cyclin-Dependent Kinase 4a
IQR	Interquartile Range
OS	Overall Survival
HRs	Hazard Ratios
CIs	Confidence Intervals
HNCs	Head and Neck Cancers
TILs	Tumor-Infiltrating Lymphocytes
HNSCC	Head and Neck Squamous Cell Carcinoma
